# Comparing the effects of individualized, standard, sham and no acupuncture in the treatment of knee osteoarthritis: a multicenter randomized controlled trial

**DOI:** 10.1186/1745-6215-14-129

**Published:** 2013-05-07

**Authors:** Eun-Jung Kim, Chi-Yeon Lim, Eun-Yong Lee, Seung-Deok Lee, Kap-Sung Kim

**Affiliations:** 1 College of Korean Medicine, Dongguk University, Gyeongju, South Korea; 2 Department of Medicine, Dongguk University, Gyeongju, South Korea; 3 College of Oriental(Korean) Medicine, Semyung University, Chung-ju, South Korea; 4 Department of Acupuncture and Moxibustion, Dongguk University International Hospital, Siksa-dong, Ilsandong-gu, Goyang, Gyeonggi-do, South Korea

**Keywords:** Acupuncture, Syndrome differentiation, Sa-am acupuncture, Individualized treatment, Knee osteoarthritis, RCT

## Abstract

**Background:**

Acupuncture is an effective yet complex therapy, integrating syndrome differentiation, selection of appropriate acupoints and skillful needling techniques. Clinicians carefully tailor acupuncture treatment to each patient. However, most clinical trials of acupuncture have been based on a standardized formula of points for every patient without properly accounting for individualdifferences and, as a result, have not been reflective of the true efficacy of clinical practice. To determine the efficacy of meridian-based syndrome differentiation and Sa-am acupuncture, we have designed a simple pragmatic trial providing individualized treatments while working within a general framework.

**Methods/Design:**

The study is designed to be a parallel, patient- and assessor-blind, randomized controlled trial (RCT). A total of250 patients with knee osteoarthritis (OA) will be recruited from two independent hospitals, Semyung University Oriental Medicine Hospital in Chung-ju and Dongguk University Oriental Hospital in Ilsan, South Korea. Patients will be randomly allocated into four treatment groups: 1. individualized, meridian-based syndrome differentiation and Sa-am acupuncture treatment;2. standard acupuncture treatment;3. sham acupuncture treatment; and 4. no acupuncture treatment. Patients in groups 1 to 3 will be treated by certified oriental medicine doctors twice a week for 6 weeks. The primary outcome measure will be the self-reported total Western Ontario and McMaster Universities Osteoarthritis Index (WOMAC) score change. The trial will also include secondary outcome measures.

**Discussion:**

This trial is designed to determine the efficacy of individualized acupuncture treatment in patients with knee OA by comparing the differences between individualized, standard, sham and no acupuncture treatments. The results of this trial may validate the efficacy of individualized acupuncture therapy, encouraging its widespread use.

**Trial registration:**

ClinicalTrials.gov: NCT01569230

## Background

Most clinical trials are designed to evaluate the efficacy and safety of medical treatment [1]. Recently, there has been increased interest in using acupuncture methods and other alternative medicines for treating diseases [2], especially those without effective conventional treatment options, such as osteoarthritis(OA) [3]. OA is one of the top ten disabilities affecting people in developed countries and significantly impacts the lives of many people [4]. At present, however, no medications are available to effectivelytreat OA. Since many patients are turning to alternative therapies [5-7], it has become necessary to determine their efficacy. For example, many clinical trials have been performed to evaluate the efficacy of acupuncture [8]. However, the results from these studieshave been criticized owing to a lack of definitive evidence regarding the efficacy of acupuncture; some studies suggest that acupuncture has clear benefits, whereas others are less conclusive [8].Many practicing oriental medicine doctors believe that this lack of definitive evidence is due to the absence of individualized syndrome differentiation from the trial design, a fundamental aspect of traditional Chinese medicine [9].

Many clinical trials on acupuncture have been explanatory in nature, emphasizing the standardization of treatments, rather than taking a pragmatic approach and compiling clinically relevant data [10]. Clinical research can be divided into two main categories, explanatory and pragmatic. Explanatory trials focus on evaluating the efficacy of a treatment in ideal, standardized environments, whereas pragmatic trials seek to determine the efficacy in clinically applicable settings [11,12]. Acupuncture therapy involves various elements unique to individual patients. For example, it must consider the signs and symptoms of each patient in differential symptom diagnosis, select the proper acupoints for a particular illness and utilize a refined needling technique [13]. In addition, to enhance the efficacy of treatment, the acupoints selected must be determined based on an understanding of each patient's individual characteristics. Hence, explanatory trials based on a single set of standardized acupoints are not appropriate for testing the efficacy of acupuncture [10].

Although pragmatic trials more closely mimic clinical settings, they also have inherent flaws. For exampletreatments often include minute variations, depending on each clinician's diagnostic methods and needling techniques, making the trials difficult to replicate [14-16]. Thus, it is necessary to enhance the reproducibility of pragmatic trials testing individualized acupuncture therapy, so that other practitioners may be better able to replicate the general techniques, while still mimicking routine clinical practice [12].

In this study, we propose to treat patients diagnosed with knee OA with anindividualized, meridian-based [17]syndrome differentiation acupuncture method. For each patient, we will select the most appropriate of the many methods available, including the Eight Principles, the Zang-fu organ system, the Qi-Blood-Yi method and the Triple Burner [18].Meridian-based syndrome differentiation was chosen because it allows the selection of specific acupoints connected to the area of pain [19-21] and considers the particular characteristics of each patient, while still being a generalized, reproducible method. Furthermore, a recent survey of oriental medicine practitioners in Korea reported that meridian-based syndrome differentiation, in conjunction with Sa-am acupuncture, is the most commonly used form of acupuncture treatment in Korean clinical practice [21].

This trial is therefore designed to assess the efficacy of meridian-based syndrome differentiation acupuncture therapy. Patients will be randomly allocated into four treatment groups: 1. individualized, meridian-based syndrome differentiation and Sa-am acupuncture treatment;2. standard acupuncture treatment;3. sham acupuncture treatment; and 4. no acupuncture treatment.

## Methods/Design

### Study design

This prospective parallel, single-blinded (assessor and patient), randomized controlled trial (RCT) is designed to assess the efficacy and safety of individualized acupuncture therapy in patients with knee OA. It will be conducted at two clinical research centers in South Korea, Semyung University Oriental Medicine Hospital in Chung-ju and Dongguk University Oriental Hospital in Ilsan, with patients divided into four parallel groups. The research protocol has been approved by the institutional review board (IRB) at each center, and each patient will be asked to provide informed consent.

### Recruitment

Participants will be recruited through advertisements in local newspapers and bulletin boards in each trial center, with a target sample size of 250 patients. All patients will be screened initially by telephone with regard to selection criteria. If appropriate, their eligibility will be determined by both an orthopedist and a radiologist through physical and radiological examinations.

### Participants

Patients will be selected from outpatients aged 40 years to 80 years with painful knee OA, as defined by the American College of Rheumatology (ACR) [22]. Patients with pain in one or both knees during the previous 3 months or longer and rated >40 mm on a visual analog scale (VAS; 0 to 100 mm) will be selected for the trial.Patients will be excluded from the study if they have: 1. trauma or surgery on the knee(s) within 6 months prior to enrollment, causing pain or functional problems; 2. a history of prolotherapy, with injections of hyaluronic acid or cortisone within the previous 3 months; 3. physical or laboratory findings indicating infection, the presence of an autoimmune disease or inflammatory arthritis; 4. serious organic disease (for example arrhythmia, angina pectoris, stroke, asthma) with resultant severe dysfunction; 5.more severe pain in regions other than the knee joint; or 6.if they are pregnant. In addition, to ensure that the results from this study are only due to the treatments from this study, all participants will be required to not have received acupuncture therapy for 2 weeks prior to entering this trial.

### Randomization and blinding

Patients will be randomized by an independent statistician using a computerized, random-number generator through the block-randomization method of SAS version 9.1(SAS Institute Inc, Cary, NC, USA) for sequence generation. Separate randomization files will be created for each of the two sites. Allocations will be concealed using sealed, opaque envelopes. After the assessor has screened the patient for inclusion and the patient has signed the informed consent form, the next envelope in the sequence will be provided, immediately prior to treatment, to the certified oriental medicine doctor who will perform acupuncture. By this method, a total of 250 participants will be randomly assigned to the four groups. Although all patients and their screening assessors will be blinded to treatment, the certified oriental medicine doctor applying acupuncture will not.

### Treatment

Patients will be randomly allocated into four treatment groups: 1. individualized, meridian-based syndrome differentiation and Sa-am acupuncture treatment;2. standard acupuncture treatment;3 sham acupuncture treatment; and 4. no acupuncture treatment. Patients in groups 1 to 3 will be treated twice weekly for 6 weeks. The trial length was based on a survey of certified oriental medicine doctors, in which 50.6% of respondents indicated that treatment for over 5 weeks is necessary to treat chronic knee pain, such as that caused by OA.

For patients with bilateral OA, the side with more pain at baseline, as assessed by a VAS, will be treated and evaluated throughout the study.

#### General acupuncture preparation and needling technique

To ensure that the study is single-blinded and that patients are not aware of the differences between treatments, we propose using a Park sham needle guide-tube [23,24] for all three acupuncture treatment groups. This will ensure that the patient will not observe any outwardly discernible differences among individualized, standard and sham acupuncture, although the penetration of the needles will differ among these three groups.

Prior to needle insertion, the area of the skin will be sterilized. Acupuncture will be performed using sterile stainless steel disposable acupuncture needles (DongBang Acupuncture Inc, Korea).The depth of insertion at each point was predetermined to be within the normal range of 8 mm to 30 mm, depending on the location of the point. Following needle insertion, the acupuncture point will be stimulated using bidirectional rotations of the needle sleeve, to achieve the sensation known as Deqi.

In patients randomized tothe individualized and standard acupuncture groups, an electroacupuncture device (STN-111,StraTek Co, Korea) will be used to provide electrical stimulation to the proximal acupoints, with the clinician adjusting the intensity so that the patientfeels an uncomfortable but not painful sensation. The needles will be kept in place for 20 minutes.

Due to ethical considerations, patients who have previously taken analgesics and non-steroidalanti-inflammatory drugs (NSAIDs) will be allowed to continue to take these medications during the study period. However, we will request that patients avoid physical therapy as much as possible to ensure that our results reflect the role of acupuncture therapy, not other forms of treatment. Patients will be questioned about having received medication at each visit and this information will be recorded for analysis of the data.

#### Individualized acupuncture

The diagnostic and treatment techniques to be used in the individualized acupuncture group were determined through interviews conducted with 234 Korean oriental medicine practitioners between 23November 2009 and 9 January 2010 (Kim *et al*., unpublished data). This survey found that the most frequently used syndrome differentiation and acupuncture method for knee pain were syndrome differentiation based on meridian theory and Sa-am acupuncture. Many oriental medicine doctors in Korea performed acupuncture on both the proximal and distal acupoints at the same time. Based on these findings, the individualized treatment method that will be used in this study is meridian-based syndrome differentiation and Sa-am acupuncture. Two types of acupoints will be selected: the proximal and distal acupoints connected to the areas of pain.

For distal acupoints,the individualized acupuncture method is based on the five elements principle in Sa-am acupuncture as well as the 12 meridian theory. According to this theory, the four causes of imbalances in meridian energies are deficiency, excess, cold and heat. Since there are 12 meridians with four possible imbalances, there are a total of 48 pre-established acupuncture prescriptions, each with five Shu transport acupoints to use to restore these imbalances [25]. Of the 12 meridians, however, only six pass through the knee. After asking the patient which part of the knee exhibits the greatest amount of pain, the certified oriental medicine doctor will determine which of the six meridians passing through the knee is closest to the source of pain and treat that meridian with the four Sa-am acupoints listed in Table 1. After inserting the needles, the practitioner will rotate the needles either clockwise or counterclockwise for tonification and sedation [26].

**Table 1:** Acupoints for individualized treatment

**Distal acupoints(four)**	**Proximal acupoints (five)**
Spleen deficiency(HT8, SP2(+), LR1, SP1(-))	Three of five acupoints(SP9, ST35, EX-LE5, BL39, EX-LE2) plus two Ashi points
Kidney deficiency (LU8, KI7(+), SP3, KI3(-))	
Liver deficiency (KI10, LR8(+), LU8,LR4(-))	
Gallbladder deficiency (BL66, GB43(+), LI1, GB44(-))	
Bladder deficiency (LI1, BL67(+), ST36, BL40(-))	
Stomach deficiency (SI5, ST41(+), GB41, ST43(-))	
One of the six possible meridians were selected for treatment	

+/-, signifies that these points have been rotated bidirectionally to stimulate tonification (+) and sedation (-).

By allowing the practitioners to choose the meridian channel based on each patient's symptoms, the proposed approach will provide patients with individualized therapy within a general, replicable framework. Before each treatment, a certified oriental medicine doctor will determine whether the patient’s clinical condition has changed; if so, the selection of acupuncture points will be reconsidered.

For proximal acupoints,in addition to using Sa-am acupuncture on the distal acupoints, proximal acupoints will be selected for each individualized treatment, since this is common practice for oriental medicine doctors in Korea. Based on the knee area in which each patient experiences pain, a certified oriental medicine doctor will choose two Ashi points connected to the area of pain, as well as three acupoints from the five popularly used acupoints:SP9(Yinlingquan), ST35(Dubi), EX-LE5(Xiyan), BL39(Weiyang) and EX-LE2(Heding). Since electrical stimulation can only be done in pairs, four of the five proximal acupoints will be stimulated electrically with a mixed wave frequency between 2Hz and 100Hz.

#### Standard acupuncture

The standard acupuncture treatment method for knee OA has already been described by Berman *et al*. [27]. The methods and materials used will be the same as those in the individualized acupuncture group, except for the selection of acupoints and electrical stimulation. Standard treatment will require the use in all patients of nine acupoints: SP9,GB34, ST36, ST35, EX-LE5 (Xiyan), BL60, GB39, SP6 and KI3.Electrical stimulation will also be applied to Xiyan knee points at a low frequency(8 Hz), with square biphasic pulses for 20 minutes, as previously described [27].

#### Sham acupuncture

Sham acupuncture treatment will be performed using a non-penetrating acupuncture device, Parksham acupuncture (DongBang Acupuncture Inc), consisting of a toothpick in a needle guide-tube, which is used to mimic needle insertion and withdrawal. Acupuncture-naïve patients find this method to be indistinguishable from true acupuncture [24], even though the needles do not penetrate the skin. Sham acupuncture will be applied to the same nine acupuncture points as in the standard acupuncture group. This Parksham acupuncture device has been used in several previous clinical research studies [28-30].

To simulate electrical stimulation, a mock-electrical stimulator (STN-111, StraTek Co)with the same appearance and sound as the conventional electroacupuncture device will be used, but without actual electrical stimulation.

#### No acupuncture treatment group

Patients in the control group will receive no acupuncture treatments, but will be assessed at each visit.

### Quality assurance

To ensure that treatments are of a high standard and delivered in accordance with the trial protocol, the qualifications and training certifications of each oriental medicine doctor will be thoroughly checked prior to the start of the trial. All treatments will be administered by practitioners with extensive clinical experience performing acupuncture for knee pain and the following qualifications:1. graduation from a 6-year, full-time course in oriental medicine, taught as a college program;2. certification by the Korean Ministry of Health and Welfare as an oriental medicine doctor;3. more than 1 year of postgraduate clinical training in an oriental medicine hospital; and 4. completion of the first-year residency program in the Department of Acupuncture and Moxibustion at Dongguk and Semyung University.

Prior to inclusion in this trial, each clinician will attend a 2-day training workshop on the methods of treatment. They will be taught to use the plastic Park sham needle-guiding tubes,provided written protocols and standardized recording documents. Since the interactions between oriental medicine doctors and participants can strongly influence the outcome of treatment, these interactions will be strictly limited, except when checking the patient’s health and reporting adverse events during the course of the trial.

In addition, to improve the quality of the study, outcome assessors will be blinded to group allocation and will not be involved in providing the interventions. To further ensure that the patients are blinded to group allocation, a cloth barrier will be placed onto each participant's neck to prevent the patient from seeing the acupuncture treatment taking place.

### Analysis of data

The primary efficacy endpoint of the study will be the change in Western Ontario and McMaster Universities Osteoarthritis Index (WOMAC) [31] score from before to after the 6-week, 12-visit trial.

The WOMAC is a widely used, reliable, valid and responsive measure of treatment outcomes in patients with OA of the hip or knee [31]. The WOMAC index consists of 24 questions (5 related to the amount of pain, 2 to stiffness and 17 to physical function), and takes less than 5 minutes to complete [32]. The Likert version of the WOMAC rates each question on an ordinal scale of 0 to 4, with lower scores indicating lower levels of symptoms or physical disability [33].

Secondary efficacy endpoints will include: 1. the success rate of treatment; 2. change in scores on the WOMAC pain subscales; 3. 100 mm pain VAS on walking; 4. results of a 6-minute walk test; and 5.investigator global assessment (IGA)/patient global assessment (PGA). Treatment success will be defined as ≥36% improvement in WOMAC score, and treatment failure by a change <36% in WOMAC score or a missing value [34].The 100 mm pain VAS on walking is used to evaluate the severity of pain. The patient will be asked to walk 15 m in a straight line and to determine his/her level of pain from 0 mm (absence of pain) to 100 mm (worst pain imaginable).The 6-minute walk test, which measures the distance a person can walk in 6 minutes, has been found to reliably measure functional exercise capacity and has been frequently used in OA-related trials [35,36]. For IGA and PGA, the oriental medicine doctor and the patient, respectively, will evaluate general improvements in OA pain-related symptoms, using a five-grade scale: excellent, good, fair, poor and aggravated. The 100 mm VAS on walking, WOMAC pain scale and IGA/PGA will be assessed at baseline, at each treatment session, and 12 weeks after the start of treatment. The WOMAC and 6-minute walk tests will be assessed at baseline, and after 3, 6 and 12 weeks from the start of the trial.

In addition, the patients will be asked to record their symptoms according to a VAS each morning and evening during the duration of the trial. They will also be asked to record the medications they take, such as analgesics and NSAIDs for knee pain, at the beginning of the trial, at each treatment session, at the end of treatment (week 6), and at 12 weeks after the start of the treatment (Table 2).

**Table 2:** Example of outcome measurements

**Measures**		**Treatment visit**												
	**Baseline**	**1**	**2**	**3**	**4**	**5**	**6**	**7**	**8**	**9**	**10**	**11**	**12**	**Follow-up**
*Primary outcome measure*														
WOMAC^a^	x							x					x	x
*Secondary outcome measure*														
Success rate^b^	x							x					x	x
WOMAC pain(subscale)	x	x	x	x	x	x	x	x	x	x	x	x	x	x
100 mm pain VAS on walking	x	x	x	x	x	x	x	x	x	x	x	x	x	x
6-minute walk test	x							x					x	x
IGA/PGA	x	x	x	x	x	x	x	x	x	x	x	x	x	x

^a^The primary outcome is the change of WOMAC score after the 12-visit, 6-week trial.^b^Success will be defined as ≥36 % improvement in WOMAC score, and failure as <36 % improvement or missing values.IGA, investigator global assessment; PGA, patient global assessment; VAS, visual analogue scale; WOMAC, Western Ontario and McMaster Universities Osteoarthritis Index.

Any reported adverse events will be recorded throughout the study, and vital signs will be monitored at each visit.

### Sample size

The primary efficacy parameter is change in WOMAC score from baseline to the end of treatment after 6 weeks. The standard deviation (SD) of the WOMAC score is 3.35 [27]. In calculating the appropriate sample size for this study, we sought to obtain a more conservative SD, with a true mean difference of 2.34.

With *μ*_1_ and *μ*_2_ representing the mean WOMAC scores for the test and control groups, respectively, the null-hypothesis

$$H_{0}:\mu_{1}-\mu_{2}=0$$

will be tested versus the alternative hypothesis

$$H_{1}:\mu_{1}-\mu_{2}\neq 0$$

Using these hypotheses, we calculated the sample size needed to achieve 80% power using the equation:

n=Zα/2+Zβ2σ2μ1-μ22.

To show that the individualized treatment group is not equal to the control groups, patients will be randomized 2:2:1:1 to the individualized treatment group, standard treatment group, sham group and untreated group, respectively. Using these assumptions and specifications, a sample size of 198 individuals (66:66:33:33) will provide a power of 80% at a significance level of 0.05. To allow for a 20% dropout rate, a slightly higher number, 250 patients, will be randomized in the trial.

### Statistical analysis plan

All statistical analyses will be performed using SAS version 9.1 or a later version. All statistical tests will be two-sided and the level of significance will be set at 0.05. The last observation carried forward (LOCF) method will be used to input data with dropouts. Continuous variables will be expressed as mean ± SD, and categorical variables as number and percentage.

The primary endpoint will be compared in the four groups by analysis of covariance (ANCOVA),with treatment means adjusted to baseline WOMAC scores as a covariate. Confidence intervals will be derived from ANCOVA. Repeated measures analysis of variance (ANOVA) will also be used to analyze primary and secondary efficacy variables. Categorical data will be analyzed using Pearson’s chi-square test or Fisher’s exact test.

## Discussion

The individualized acupuncture treatment method proposed in this study, consisting of meridian-based syndrome differentiation and Sa-am acupuncture, is not only the most commonly method used by certified oriental medicine doctors in Korea to treat patients with OA [21,25], but can be generalized for widespread use in the future. In this trial, we will evaluate the efficacy of this individualized treatment method by comparing it with standard, sham and non-treatment groups.

The acupuncture methods employed in this study are the closest to actual clinical practice and can be used in future studies. Moreover, the techniques of meridian based and Sa-am acupuncture that we will use in this study are based on the consensus of many certified oriental medicine doctors in Korea. In addition, we will strive to conduct a high quality, single-blinded clinical trial by ensuring that the assessors and participants are not informed of the type of treatment, by using a variety of controls including Park sham acupuncture, and providing trial-specific training of the exact methods used to well-qualified and certified oriental medicine doctors.

In conclusion, this research involves three different controls, and it will help elucidate the efficacy of individualized acupuncture versus standard, sham and no acupuncture groups.

### Trial status

Participant enrollment is still being undertaken. Enrollment and trial completion is expected to be finished by the end of 2013.

## Abbreviations

ACR: American College of Rheumatology; ANCOVA: Analysis of covariance; ANOVA: Analysis of variance; IGA: Investigator global assessment; IRB: Institutional review board; LOCF: Last observation carried forward; NSAID: Non-steroidal anti-inflammatory drug; OA: Osteoarthritis; PGA: Patient global assessment; RCT: Randomized controlled trial; SD: Standard deviation; VAS: Visual analogue scale; WOMAC: Western Ontario McMaster Universities Osteoarthritis Index.

## Competing interests

The authors declare that they have no competing interests.

## Authors’ contributions

KSK, SDL, EYL and EJK conceived of the project, and formulated and coordinated its performance. EJK wrote the first draft of the manuscript. CYK provided statistical advice and wrote the relevant sections of the manuscript. KSK and SDL revised the manuscript. All authors gave final approval of the version to be submitted.
